# Effortless inhibition: habit mediates the relation between self-control and unhealthy snack consumption

**DOI:** 10.3389/fpsyg.2014.00444

**Published:** 2014-05-16

**Authors:** Marieke A. Adriaanse, Floor M. Kroese, Marleen Gillebaart, Denise T. D. De Ridder

**Affiliations:** Department of Clinical and Health Psychology, Utrecht UniversityUtrecht, Netherlands

**Keywords:** self-control, habit, health, inhibition, effortless, snacking

## Abstract

In contrast to prevailing beliefs, recent research suggests that trait self-control promotes health behavior not because those high in self-control are more successful at resisting single temptations, but rather because they develop adaptive habits. The present paper presents a first empirical test of this novel suggestion by investigating the mediating role of habit in explaining the relation between self-control and unhealthy snacking behavior. Results showed that self-control was negatively associated with unhealthy snack consumption and unhealthy snacking habits. As hypothesized, the relation between self-control and unhealthy snack intake was mediated by habit strength. Self-control was not associated with fruit consumption or fruit consumption habits. These results provide the first evidence for the notion that high self-control may influence the formation of habits and in turn affect behavior. Moreover, results imply that self-control may be particularly influential in case of inhibiting unhealthy food intake rather than promoting healthy food intake.

## INTRODUCTION

Self-control is associated with a variety of positive outcomes, including academic performance (Duckworth and Seligman, 2005), adjustment (Tangney et al., 2004), and health behavior (Hofmann et al., 2008). Regardless of the specific behavioral domain that is involved, adaptive outcomes of self-control are considered to result from the ability to withstand impulses for immediate gratification of one’s needs in view of one’s long term-goals (Baumeister et al., 1998). Though self-control can be conceptualized as a state as well as a trait (Tangney et al., 2004), in the current paper we focus on the latter. In particular, building on recent insights (De Ridder et al., 2012; Hofmann et al., 2012), we aim to investigate the mediating role of habit strength in order to gain more insight into the mechanisms by which trait self-control results in beneficial outcomes.

Although self-control has often been a topic of investigation, and researchers agree that it generally has beneficial effects on a variety of behaviors, *how* it operates remains relatively unclear. Still, “researchers agree that self-control focuses on the efforts people exert to stimulate desirable responses and inhibit undesirable responses (De Ridder et al., 2012, p. 77),” implying that exerting self-control is effortful and involves the active self (Baumeister et al., 1998). In a similar vein, (Fujita (2011, p. 355) noted that many scholars “explicitly or implicitly define self-control as the effortful inhibition of impulses.” This view of self-control as effortful is not restricted to trait self-control, but is also apparent in the widely used ego-depletion paradigm, where (state) self-control is, similar to a muscle, theorized to get depleted after using it, obviously implying that exerting of self-control is effortful (Muraven and Baumeister, 2000).

However, in contrast to the common conception of self-control as an effortful process, a recent meta-analysis examining the effects of self-control demonstrated that the beneficial effects of self-control may not necessarily be the result of an effortful inhibition of undesirable responses or initiation of desired responses (De Ridder et al., 2012). That is, contrary to the authors’ expectations, results showed that the effects of self-control were larger for habitual behaviors than for behaviors under effortful control (De Ridder et al., 2012). Both in case of desired behaviors and undesired behaviors, the effect of self-control on behaviors that were rated to be mostly effortful (i.e., that required conscious intention or deliberation, such as solving anagrams or making plans) was almost 2.5 times lower than on behaviors that were rated to be mostly automatic (e.g., addictive or habitual behaviors).

This finding puts the beneficial effects of self-control in a new perspective as this implies – somewhat counter intuitively – that people with high self-control are more successful because they use their self-control less frequently as a result of having established effective habits or routines, rather than being more successful in resisting single temptations (for a similar line of reasoning see Fujita, 2011). Some more evidence for this suggestion was recently provided by Hofmann et al. (2012) who showed that people with high self-control reported weaker desires, less motivational conflict, and lower levels of resistance toward a wide range of desires. Importantly, findings also showed that people with high self-control encountered fewer desires that were rated by others as* problematic*, ruling out the possibility that people high in self-control merely fail to acknowledge the motivational conflict associated with their desires. These findings suggest that people with high self-control are more successful than people with low trait self-control at avoiding problematic desires, implying, in line with De Ridder et al. (2012) that self-control may be particularly related to the forming of adaptive routines or habits rather than the ability to control oneself in specific situations.

As no direct evidence as of yet exists, the present paper aims to provide a first empirical test of the suggestion that self-control operates through establishing adaptive habits. By habits we refer to “learned sequences of acts that have become automatic responses to specific cues, and are functional in obtaining certain goals or end-states” (Verplanken and Aarts, 1999, p. 104). Note that this means that, in line with Verplanken and Orbell (2006), in the present manuscript habit is considered a psychological construct, that extends beyond past behavioral frequency to include features of automaticity and goal-directedness. That is, we view habits as mental associations between situational cues and behavior that have developed through repetition to the extent that the behavior follows *automatically* (Bargh and Gollwitzer, 1994; Aarts and Dijksterhuis, 2000; Verplanken, 2006), or, without awareness, unintentionally, efficiently, and with very limited controllability (Bargh, 1994), upon encountering the situational cue.

Since eating behavior is often portrayed as a typical self-control dilemma, and previous research has suggested that food intake is the prototype of a behavior that is highly influenced by the ability to exert self-control (e.g., Hofmann et al., 2007; Kuijer et al., 2008; Nederkoorn et al., 2010), eating behavior was chosen as the domain to investigate our hypothesis. We chose to assess snack intake as a specific type of eating behavior in order to allow for reliably assessing the degree to which it occurs habitually, using the self-report habit index (SRHI; Verplanken and Orbell, 2006; currently the most widely used scale for assessing habit strength). Moreover, snacks are a food type that is likely to be impacted by self-control for the participants in our study, as students frequently have more choice over their snacks than their meals as many students eat meals prepared by others (e.g., roommates or parents). Finally, targeting snack consumption is relevant from a health perspective as unhealthy snacks have been found to be an important contributor to overweight and obesity (Duffey and Popkin, 2011).

The current study incorporates both unhealthy snack intake as well as fruit consumption. Importantly, we expect to find an effect of self-control on unhealthy snack intake but not on fruit consumption, as eating more fruits – in contrast to resisting unhealthy snacks – is typically not considered a self-control dilemma. That is, while unhealthy snacks reflect a clear trade-off between tastiness and healthiness, in previous research students typically rated fruits as both healthy and tasty demonstrating that eating more fruits should not involve a dilemma (Salmon et al., 2014). In addition, we chose to focus on fruits rather than healthy snacks in general to increase the chance that indeed two separate behaviors where targeted. That is, eating fruits has health benefits and may thus represent a goal in itself, whereas an increase in healthy snacks does not have any health benefits in its own right and, may thus, rather than representing a separate goal, be a reflection of the goal to eat fewer unhealthy snacks (i.e., to substitute unhealthy snacks by healthy alternatives).

In the present study, measures of habit strength related to both unhealthy snack intake and fruit intake will be included alongside measures of self-control. In case self-control indeed predicts unhealthy snack intake and/or fruit intake, mediation analyses will be conducted to test whether the effects of self-control are mediated by habit strength.

## MATERIALS AND METHODS

### PARTICIPANTS AND PROCEDURE^1^

To ensure that limiting unhealthy snacks indeed involves a self-control dilemma for our participants, we only include people who indicate that they are trying to eat healthily (i.e., for whom unhealthy snacks indeed involve a trade-off between tastiness and the goal to eat healthily). Undergraduate students who responded affirmatively to the question “Are you trying to eat healthily?” (*N* = 87) were invited to fill out a questionnaire on eating behavior assessing several psychological predictors of snack intake, and then to keep a snack diary for 7 days. The measures in the questionnaire that were used for the purpose of the present study were intention to limit unhealthy snack intake/eat sufficient fruits, dispositional self-control, and habit strength of fruit and unhealthy snack consumption. Eighty two participants completed the entire study (i.e., drop out is 5.7%), for which they received €5 reimbursement. Three participants who seemed to have included meals in their snack diary or who did not fill out the diary correctly were excluded. In addition, checking for outliers (>2.5 SD) on the dependent measures resulted in exclusion of two participants; one participant who consumed an extreme amount of unhealthy snacks (1205 kcal on unhealthy snacks per day) and one participants who consumed an extreme amount of fruits (4.4 pieces of fruit per day). After exclusion of these participants, the final sample consisted of 77 participants. The majority of these participants was female (92%). Participants were between 17 and 31 years of age (*M* = 21.03, SD = 2.77) and had an average body mass index [BMI; weight/(height^2^)] of 22.11 (SD = 3.31). Four participants did not indicate their weight.

### QUESTIONNAIRE

Intention was measured for eating sufficient fruit and limiting unhealthy snack intake separately. For both fruit and unhealthy snack intake, three items were included (“I intend/plan/want to…”) that assessed the intention to eat two portions of fruit per day (Cronbach’s α = 0.93) or the intention to limit one’s snack consumption (Cronbach’s α = 0.96) in the coming week on 7 point scales ranging from 1 (totally disagree) to 7 (totally agree).

Dispositional self-control was assessed using the brief Self-Control Scale (Tangney et al., 2004) which includes thirteen items (e.g., “I am good at resisting temptation”) that are answered on 5-point scales ranging from 1 (*not at all applicable to me*) to 5 (*very much applicable to me*), Cronbach’s α = 0.81). After recoding reverse coded items, a higher score reflects more self-control.

To assess the habit strength of eating fruits and unhealthy snacks two adapted versions of the SRHI (Verplanken and Orbell, 2006) were administered. The SRHI consists of 12 items measuring habit strength in terms of repetition and automaticity. For the purpose of this study, the SRHI was adapted in such a way that it included 12 items which referred to the habit of eating unhealthy snacks (α = 0.95) and 12 items which referred to the habit of eating fruits (α = 0.95; e.g., “Eating unhealthy snacks/fruits is something I do without thinking about it”). Participants indicated their responses on 7-point scales ranging from 1 *(totally disagree*) to 7 *(totally agree).*

### SNACK DIARY

After completing the questionnaire, participants received the snack diary (adapted from Adriaanse et al., 2009). Snacks were defined as any healthy or unhealthy foods eaten in between meals. The snack diary was thoroughly explained by the experimenter, and included instructions and an example of a diary entry on the first page. Participants were requested to record the amount of snacks they consumed each day for seven consecutive days. Each of the seven entries (one for each day) consisted of one column with 12 categories of unhealthy snacks (e.g., candy bars, crisps) and one column with thirteen categories of healthy snacks (e.g., fruit, rice crackers) each with standardized portion sizes (e.g., “handful” for crisps). Categories and portion sizes were based on advice from a registered dietician. For both healthy and unhealthy snacks an “other” option was also provided. For the present study, we were interested in the consumption of unhealthy snacks as well as the consumption of fruits. In the analyses, we chose to focus on fruits instead of all healthy snacks, as eating fruits is most typically associated with actual health benefits, in contrast to for example rice crackers that are generally considered a healthy alternative to fatty or sugary snacks, but that do not have a beneficial effect on health *per se*^2^.

Based on the diary entries the amount of unhealthy snacks and the amount of fruits consumed during the 1 week period were calculated. As the unhealthy snacks that participants consumed varied considerably in size and calories, unhealthy snack consumption was expressed in kilocalories (kcal). The number of kcal derived from unhealthy snacks was calculated by multiplying each standard amount of unhealthy snacks consumed by the average amount of kcal it contained (based on guidelines from the Dutch Nutrition Centre and checked by a dietician). For fruits, in terms of health benefits the number of servings was deemed more relevant than calories – moreover, fruits varied little in size and calories – so the consumption of fruits was expressed in servings (e.g., one serving equals one banana or one serving of grapes; cf. Adriaanse et al., 2009, 2010).

## RESULTS

Participants reported in the snack diary to have consumed 2634 (SD = 1403) kcal on unhealthy snacks (approximately 376 kcal a day) and 8.5 servings of fruit (SD = 6.1; approximately 1.2 serving of fruit a day) over the entire week. Participants reported moderately strong unhealthy snacking habits (*M* = 3.95, SD = 1.36) as well as moderately strong fruit consumption habits (*M* = 3.89, SD = 1.45). The means and correlations of the variables under study are reported in **Table 1**. Before discussing the key correlations between snack intake, habit, and self-control, it is noteworthy to mention that BMI was positively related to fruit consumption (*r* = 0.28, *p* = 0.02), it was not significantly correlated with unhealthy snack intake, (*r* = -0.17, *p* = 0.16), or with self-control, (*r* = 0.04, *p* = 0.74).

**Table 1:** Means, standard deviations, and correlations.

	1	2	3	4	5	6	7	8	9	10
Sex (1)	–									
Age (2)	0.19	–								
BMI (3)	0.06	0.18	–							
Self-control (4)	-0.10	-0.16	0.04	–						
Intention unhealthy snack (5)	-0.20	-0.23*	0.13	0.10	–					
Intention fruit (6)	-0.27*	-0.31**	0.14	0.03	0.33**	–				
Habit unhealthy snack (7)**	-0.02	0.13	0.02	-0.26*	-0.13	-0.08	–			
Habit fruit (8)	-0.29*	-0.21	0.27*	0.18	0.09	0.45**	-0.20	–		
Unhealthy snack intake [Kcal] (9)	0.19	-0.08	-0.17	-0.30**	-0.21	-0.33**	0.38**	-0.30*	–	
Fruit intake (pieces) (10)	-0.19	-0.06	0.28*	0.12	0.25*	0.46**	-0.05	0.57**	-0.17	–

*M*	92%^1^	21.03	22.11	2.98	4.05	4.34	3.95	3.89	2634	8.5
SD	–	2.77	3.31	0.57	1.90	1.93	1.36	1.45	1403	6.1

^1^percentage female; *p < 0.05; **p < 0.01.

Correlations between the key variables showed that self-control was significantly related to unhealthy snack intake (*r* = -0.30, *p* = 0.01) and unhealthy snacking habits (*r* = -0.26, *p* = 0.03), but not to fruit intake (*r* = 0.12, *p* = 0.29)^3^; or fruit consumption habits (*r* = 0.18, *p* = 0.13). Unhealthy snack intake was positively related to unhealthy snack habit strength, (*r* = 0.38, *p* <0.01) and negatively related to fruit consumption habits (*r* = -0.30, *p* = 0.01). Fruit consumption was associated with fruit consumption habits (*r* = 0.57, *p* < 0.01), but not with unhealthy snacking habits (*r* = -0.05, *p* = 0.70).

The significant correlations between (a) self-control and unhealthy snack intake, (b) self-control and unhealthy snack habit strength, and (c) unhealthy snack habit strength and unhealthy snack intake suggest that unhealthy snack habit strength might mediate the relation between self-control and unhealthy snack intake. In order to formally test mediation, a bootstrapping approach was used according to the guidelines and macro developed by Preacher and Hayes (2004). Requesting 5000 bootstrapping samples (*z* = 5.000), the indirect effect between self-control and unhealthy snack intake through unhealthy snack habit strength was estimated at -201.75. The 95% confidence interval of the estimated indirect effect did not include 0 (C.I.: -522.9, -39.2), indicating that the proposed mediation was significant^4^.

As, conform our expectations, there was no significant correlation between self-control and fruit intake or fruit consumption habits, there was no basis for further analyses testing whether fruit consumption habit strength mediated the relation between self-control and fruit intake.

## DISCUSSION

The present paper aimed to shed light on the underlying mechanism of self-control. Results of the present study provided initial evidence for the recently proposed hypothesis that habits mediate the relation between self-control and behavior. That is, our results indicated that people high in trait self-control were more likely to have weaker unhealthy snacking habits, and in turn consumed less unhealthy snacks. These findings imply that at least part of the reason why people with high self-control are more successful in the self-regulation of their behavior may be that they do not put themselves in the position where they have to resist temptations often as they are more likely to prevent the creation of maladaptive habits (De Ridder et al., 2012; Hofmann et al., 2012). This implies that equating self-control with effortful inhibition as is frequently done (cf. Fujita, 2011) might not be correct as self-control may operate more by creating effective routines that are executed relatively automatically rather than by effortfully inhibiting immediate impulses once confronted by them.

While self-control was related to less unhealthy snack intake and weaker unhealthy snacking habits, there were no associations between self-control and fruit intake or fruit consumption habits. This was also in line with our predictions as we expected fruit intake to represent less of a self-control dilemma than limiting unhealthy snack intake. Students in general find fruits attractive and eating more of something that is considered healthy but also attractive should not represent much of a self-control conflict (Salmon et al., 2014). Indeed, in the present sample the intention to consume sufficient fruits was significantly related to fruit consumption habits and fruit intake, whereas the intention to limit unhealthy snack intake was not related to unhealthy snacking habits or unhealthy snack intake. This confirms the notion that the motivation to eat sufficient fruits is more easily translated into supporting routines and actual behavior, not requiring much self-control, whereas motivation to limit unhealthy snack intake is not sufficient to affect actual unhealthy snack intake and high trait self-control is required to be successful.

Some surprising (lack of) correlations with BMI are worth mentioning. First, self-control was not related to BMI in the present sample. One reason may be that we included a relatively homogenous sample of rather healthy (weight) participants.

Moreover, while BMI was positively related to fruit intake, it was not related to unhealthy snack consumption. This finding is difficult to explain. Although it could also be related to our relatively restricted sample, this is unlikely as these results are in line with correlations between BMI and fruit and unhealthy snack intake in another study in a large representative sample (*N* = 1292) of Dutch adults (Adriaanse et al., under revision).

The present findings also suggest that self-control operates mostly through the avoidance or breaking of maladaptive habits rather than the creation of adaptive habits as self-control was only (negatively) related to unhealthy snack habit strength and not to fruit consumption habits. This would be in line with the suggestion by Hofmann et al. (2012) who proposed that people with high trait control are successful at avoiding tempting situations. However, note that future research is required to investigate whether this results holds specifically for snack intake or the eating domain, or whether this applies also to other self-control dilemma’s.

In addition to shedding more light on the working mechanism of self-control, the present findings also help to explain the recent meta-analytical finding that self-control is only a weak predictor of eating behavior (De Ridder et al., 2012). This latter finding is difficult to consolidate with the fact that eating is typically used as a prototypical case to demonstrate the imperative role of self-control. However, the present findings clearly indicate that it is crucial to distinguish between unhealthy snack consumption and healthy snack consumption in studies on self-control and eating behavior as the correlation between self-control and unhealthy snack intake was significant and approximately 2.5 times stronger than the (insignificant) association between self-control and fruit intake. This suggests that, as expected, the predictive value of self-control on food intake may become larger when specifically considering unhealthy or undesired food intake. In addition it illustrates that, apparently, withholding oneself from giving in to impulses such as eating unhealthy snacks does not automatically result in striving toward valued goals (e.g., eating fruits).

Several limitations of the present study have to be noted. First of all, we included a sample of young, mostly female students with relatively healthy weights, which limits the generalizability of the present findings. Although meta-analytical evidence indicates that, in general, the effect of self-control is equally strong for males and females indicating that our unequal gender distribution should not pose a big problem, this same meta-analysis also suggests that the relatively young age and student status of our sample may have influenced our findings (De Ridder et al., 2012). Specifically, the effect of self-control is generally larger amongst younger samples which suggests that the actual relation between self-control and food intake might be weaker in a more age diverse sample. The effects of student status, however, tends to show the opposite pattern as the beneficial effects of self-control are usually smaller amongst student samples compared to community samples (De Ridder et al., 2012). All in all, it is therefore unlikely that the present results are largely inflated compared to when a more representative sample would have been recruited.

Secondly, our study relies on self-report measures only, which may limit the reliability of our assessments. Particularly with respect to the self-reporting of eating behavior, it is known that people tend to underreport their actual food intake (Klesges et al., 1992; Stice et al., 2004). Although we specifically chose to employ a previously validated food diary over a period of 1 week, which still is one of the most sophisticated measures of assessing food intake (De Castro, 2000) and most likely more accurate and reliable as compared to retrospective measures such as food frequency lists, we acknowledge that in reality the amounts of unhealthy snacks consumed may be higher than suggested by our current findings. However, we do not suspect that this would impact the relations between self-control and eating behavior that were investigated in the current paper, leaving our conclusions valid.

Third, the present data is correlational and cross-sectional (although food intake was assessed for 7 days after the measures of self-control and habit) and therefore does not allow for strict causal conclusions. Although our findings suggest a causal chain of events with higher self-control leading to less unhealthy habits and in turn a lower unhealthy snack consumption, this hypothesis needs to be further confirmed in an experimental and/or longitudinal study. Finally, our study was restricted to snack consumption only. Future research should include overall measures of food intake, including main meals, in order to gain more insight into the impact of self-control on food intake at large. In addition, such studies could include assessments of the presence of others and the degree to which people experience conflict (c.f. Hofmann et al., 2012) and perceive the snack to be (un)healthy in order to further disentangle the relation and dynamics between self-control, habit and overall food intake.

In conclusion, notwithstanding the above outlined limitations, our study provides novel insights into the underlying mechanisms of self-control in the domain of eating behavior. We showed that self-control predicts unhealthy snack consumption but not fruit consumption. Most importantly, however, results indicated that the relation between self-control and unhealthy snack consumption is mediated by habit strength, empirically supporting recent suggestions in the literature that self-control affects behavior through an automatic rather than an effortful route. These insights contribute to a new perspective on how self-control affects behavior, which appears to be less straightforward than previously assumed. Future research should examine in more detail in what way high self-control contributes to the formation of habits.

## Conflict of Interest Statement

The authors declare that the research was conducted in the absence of any commercial or financial relationships that could be construed as a potential conflict of interest.

## Footnotes

1 This study was conducted in the Netherlands. In the Netherlands, scientific research involving human subjects must undergo a review of an institutional review board if the research is subject to the Medical Research Involving Human Subjects Act (WMO). Research involving human subjects only falls within the remit of the Act if it involves any form of invasion of participants’ integrity. According to the national guidelines our study is not invasive of the participants integrity, and hence not subject to the WMO.

2 Despite our focus on fruits in the analyses, we chose to include other healthy snacks as well in the diary rather than including only fruits as health snacks. This was done to create a complete overview of participants’ snack intake and thus to allow for checking whether snacks were correctly categorized.

3 Note that the correlation between self-control and overall healthy snack consumption was also insignificant, *r* = -0.06, *p* = 0.63.

4 Based on a suggestion by one of the reviewers we also tested the reverse mediation model from self-control to habit through unhealthy snack intake. Results indicated that this mediation model was also significant with an indirect effect of -0.24, as the 95% confidence interval of the estimated indirect effect did not include 0 (C.I.: -0.56, -0.04). Although habit is indeed a consequence of behavioral repetition, still this latter mediational model is the less likely option as it generally takes more than 7 days (e.g., 18–254 days according to Lally et al., 2010) to create a habit. Moreover, in the current study the habit strength index was administered before filling out the diary making the reverse path methodologically unlikely. One reason then, for why the reverse path is also significant, may be that the snack intake reported in the diary is in fact a very accurate reflection of participants’ habitual snack consumption (we thank one of the reviewers for this suggestion).
